# The impact of brand trust on consumers’ behavior toward agricultural products’ regional public brand

**DOI:** 10.1371/journal.pone.0295133

**Published:** 2023-11-30

**Authors:** Qiyun Liu, Xinyuan Wang

**Affiliations:** School of Law and Economics and Management, Hulunbuir University, Hulunbuir, China; SGH Warsaw School of Economics: Szkola Glowna Handlowa w Warszawie, POLAND

## Abstract

The importance of agricultural products’ regional public brands, owned by relevant organizations and jointly used by several agricultural production and operation entities, is increasing in contemporary marketing research. Based on a survey of 544 consumers, this study investigates the influence of brand trust, attitude, subjective norms, and perceived behavioral control on their purchase intention and behavior toward agricultural products’ regional public brand. Through SEM, we find that brand trust toward agricultural products’ regional public brand positively influences consumer attitude, purchase intention, and purchase behavior. In addition, attitude and purchase intention mediate the relationship between brand trust and purchase behavior. While attitude and perceived behavioral control positively affect purchase intention, no similar effect is found for subjective norms. Moreover, multigroup invariance tests demonstrate that consumer behavior can be influenced by factors such as gender, age, monthly income, marital status, previous visits to the region, and purchase purpose. We therefore recommend that to bolster competitiveness, regional public brand stakeholders maintain agricultural product quality, ensure reliable purchasing and transportation channels, and enhance brand trust.

## 1. Introduction

Food, as a paramount necessity, plays a crucial role in everyone’s daily life. Everyone makes daily decisions on food purchases [1], weighing factors such as price, quality, and nutritional content. The evolution of the e-commerce and logistics sectors has broadened consumer food choices in terms of origin and variety while enhancing convenience and the shopping experience [2, 3]. Live e-commerce platforms facilitate direct interaction between consumers and agricultural product producers, increasing transparency [4]. Moreover, the COVID-19 pandemic has influenced people’s dining habits, with many opting to cook at home rather than dine out, increasing the demand for high-quality ingredients. Additionally, consumers are opting for online food purchases, signifying a shift in their behavioral patterns [5]. Such consumer behavior will not change for some time [6]. Following the emergence of consumer upgrading and the experience economy, consumers are now more concerned about the quality and safety of food and their service experience [7]. In response to competitive pressures, the Chinese government and producers have endeavored to imbue their agricultural products with unique attributes for distinction in the marketplace. In 2017, China’s Central Government Document No. 1 first called for developing agricultural products’ regional public brands. This initiative was further progressed in 2019, when the central government commenced the collection of well-known regional public brands [8]. Thus, the emergence of regional public brands for agricultural products represents a progression in marketing [9], reflecting agricultural modernization and the improved marketability of such brands.

The notion of agricultural products’ regional public brand has not yet received a strict or unified professional definition or conceptualization. Scholars have thus far used the terms regional branding [10, 11] or place branding [12, 13]. Hankinson (2010) [14] has argued that the widening domain of place branding includes destination, nation, regional, and city branding. In this study, the concept of agricultural products’ regional public brand is primarily derived from relevant documents of China’s Ministry of Agriculture and is characterized by several distinctive features. First, a regional public brand relies on a region’s specific natural ecological environment and historical and human factors; it is an intraregional industrial cluster, which serves as its regional brand identity [15]. Second, brands are owned by relevant organizations and jointly used by several agricultural production and operation entities [16], distinguishing them from corporate brands. Typically, the unique characteristics of a regional public brand are its endorsement by the public, establishment via governmental initiatives, and exclusivity [17]. Finally, similar to products and services, geographical locations can be branded [18]. Therefore, agricultural products’ regional public brands often follow an “origin name + product name” format [19]. They encompass various agricultural products including grains, oils, livestock, poultry, seafood, fruits, vegetables, traditional Chinese medicine herbs, edible fungi, tea, and forest specialties. Each region also creates its own distinctive brand based on its unique circumstances, thereby differentiating its products from similar agricultural products. Regional public brand development also enhances the quality certification standards of specific agricultural products, streamlines production methods, and extends the product’s industrial chain. The ultimate aim of agricultural products’ regional public brand is to increase consumer recognition, market competitiveness, and overall value. This approach allows agricultural producers to reap more industry profits, supporting the execution of rural revitalization strategies [20, 21].

The Chinese government’s extant certifications of agricultural products include hazard-free, green, and organic food. Combined with geographical factors, such certification is a geographical indication [22]. However, while geographical indications and regional public brands are both influenced by regional aspects, they represent distinct concepts. A regional public brand is driven by market operation, whereby consumer and market response determine its establishment; registration or certification are not prerequisites, yet they are commonly pursued by brand owners [10]. Geographical indications, in contrast, are legal constructs shielded from infringement and counterfeiting by trademark law, applied to all products within a region [23]. Nevertheless, regional public brands that integrate unique geographical conditions, production methods, and brand equity tend to hold the most significant competitive advantage in the marketplace.

The literature on agricultural products’ regional public brands has focused on brand building [24, 25] and brand integration performance [26], and some scholars have conducted case studies [16]. Concerning consumers’ perceptions of agricultural products’ regional public brands, research has shown that regional brands have failed to play their expected role and that consumers are not enthusiastic about these brands [11, 27]. In China, the market share of green agricultural products remains limited due to consumers’ inadequate understanding of these products, pricing concerns, and lack of convenient purchasing channels [2]. The literature has examined the impact of Green brand experiential quality, brand experience, brand experiential risk, and brand equity on purchase intention. However, it has provided insufficient indications as to why consumer responses following brand development fail to meet expectations [28]. Currently, comprehensive research is lacking on the complete chain of consumers’ trust in regional public brands influencing their intention to purchase, which then translates into actual buying behavior.

China has established many successful agricultural products’ regional public brands. The China Agricultural Brand Research Center at Zhejiang University, led by Hu Xiaoyun, has developed a model for assessing the value of China’s agricultural products’ regional public brands. Since 2010, this team has conducted a special assessment of China’s regional public tea brands’ value; its data shows that the value of certain tea brands has doubled [29, 30]. However, solely focusing on success stories may obscure regional public brand-building challenges and the factors impacting consumer choices. By exclusively focusing on successful case studies, we risk neglecting a key research gap: the identification of potential pitfalls in regional public brand-building and the elements consumers genuinely value. Additionally, the lack of research on less developed regional public brands underscores the urgency for thorough investigations in this field.

Therefore, this study examines the regional public brand of Hulunbuir’s agricultural products, situated in China’s Inner Mongolia Autonomous Region, which currently lacks discernible brand advantages. Occupying approximately 2.5% of China’s total land area, Hulunbuir serves as a significant nexus for grain production and animal husbandry [31]. Hulunbuir hosts eight regional public brands, each representing unique advantages and challenges. Its geographic location creates potential for brand expansion, whilst simultaneously adding complexity to the growth of its agricultural product brands. The widespread distribution of producers introduces transportation challenges, such as transport time, cost, and product freshness, which significantly influence consumer decisions. The expansion of courier services like SF Express and EMS has helped to address these challenges. Furthermore, Hulunbuir’s regional public brands focus mainly on primary agricultural products, resulting in a lesser contribution to the region’s economic value. Compared to brands in other Inner Mongolian regions, this highlights that consumer behavior towards Hulunbuir’s regional public brands necessitates further study.

This study aims to address the following questions. First, we examine the influence of brand trust toward agricultural products’ regional public brands on consumers’ attitudes, purchase intentions, and purchase behaviors.

Second, we integrate brand trust with the Theory of Planned Behavior (TPB), a fundamental approach to understanding consumer behavior [32, 33]. Our focus is to discern how the components of the TPB—namely attitudes, subjective norms, and perceived behavioral control—influence the purchase intention and behavior toward agricultural products’ regional public brand. In particular, we discuss how subjective norms can influence purchase intentions and behavior through attitudes.

Thirdly, we explore the transformation of purchase intention towards regional public brands of agricultural products into actual consumer buying behavior. This investigation is driven by the ultimate goal of every regional public brand, which is to stimulate tangible consumer purchasing activity.

This study therefore contributes to the literature in several ways. First, building on the positive influence of brand trust in purchase behavior [34], and addressing the limitations of the TPB [35, 36], we designed a framework to systematically study the factors influencing consumer intention and behavior toward agricultural products’ regional public brand.

Second, this study specifically focuses on the Hulunbuir’s agricultural products’ regional public brand. This region, despite being rich in resources, has underdeveloped regional public branding, making the wider application of our research results more significant. The data were obtained through a survey questionnaire, resulting in 544 valid responses. We also conducted path analysis via structural equation modeling (SEM) to examine the relevant direct and indirect effects.

Third, we conducted a multigroup invariance analysis to determine whether consumer characteristics influence brand trust, purchase intention, and behavior toward regional public brands. Subgroup analysis factors included gender, age, monthly income, marital status, family size, previous visits to Hulunbuir, and purchase purpose. Accordingly, this study provides empirical insights into consumer decision-making for regional agricultural brands and offers practical recommendations for brand development strategies.

The remainder of this research is organized as follows: Section 2 presents the theoretical framework and research hypotheses. Section 3 describes the research design, including scale and item development. Section 4 describes the measurement model, the results in terms of the hypotheses, and the multigroup analyses. Section 5 offers a discussion and implications of the findings. Finally, Section 6 provides the conclusion, which includes a summary of the findings, limitations of the study, and directions for future research.

## 2. Literature review and hypotheses

### 2.1 Brand trust, attitude, purchase intention and behavior

A brand is the name, term, design, symbol, or any other feature that differentiates the goods or services of one seller from those of others; a specific location or region can also establish a distinct brand identity [37]. Similar to corporate brands, regional public brands move through stages of brand evaluation, which are related to brand infrastructure, brand articulation, and communication [38]. Brand trust can be influenced by marketing strategies and brand image [39]. As such, agricultural products’ regional public brands frequently demonstrate an enhanced brand image, particularly when they garner governmental endorsement and are backed by effective marketing strategies. These products offer high-quality assurances; they originate from a unique geographical origin, a conducive natural production environment, use standardized production and processing methods, and offer visually appealing packaging, all of which serve as emblems of health and nutrition [40].

Brand trust, a cornerstone of commercial success [41] and a prerequisite for brand loyalty [42, 43], not only forms the foundation of consumers’ positive perceptions [44], but also bolsters favorable attitudes towards regional public brands. Through a regional public brand’s identity, consumers differentiate branded from generic products [45]; thus, this brand identity, along with trust, shapes customer satisfaction and purchase intention [46, 47]. Indeed, studies have shown that brand trust stimulates word-of-mouth marketing and drives consumption [48]. A case study in Beijing’s pork market has echoed this finding, highlighting brand trust’s positive role in shaping purchase intention, particularly for traceable branded products [49].

The TPB is an important theory for predicting consumer behavior patterns and an extension of the theory of reasoned action rational behavior (TRA) [50, 51]. The TPB has been widely applied to explain various consumer behavioral choices [52], with a pronounced relevance in the domain of food consumption [53, 54]. Behavioral intentions are the direct antecedents of behavior and exert control over behavioral attitudes, subjective norms, and perceived behaviors. These determinants are derived from beliefs about the possible consequences of behavior (attitude), thoughts about the normative expectations of significant others (subjective norms), and the factors controlling behavioral execution (perceived behavioral control).

Attitude refers to the degree of favorable or unfavorable evaluation of relevant behavior [55]. Attitude is a function of the perceived results of behaviors and a psychological emotion conveyed by consumer evaluations. If an attitude is positive, the behavioral intention will be more positive. According to Paul et al. (2016) [54], attitude is the strongest predictor of the willingness to purchase agricultural products. Nonsensory factors such as brand and price may also significantly impact consumers’ perceptions of functional foods [56]. Moon et al. (2022) [57], incorporating the technology acceptance model (TAM), have discovered that factors such as food quality (i.e., healthiness, hygiene, and organic nature) and brand trust significantly enhance consumers’ willingness to pay in the cafe industry. Moreover, given that regional public brands are government-led, this inherently bolsters consumers’ confidence in product quality, amplifies their positive attitudes, and ultimately stimulates their purchase intentions [58], even prompting actual purchases. Accordingly, we posit the following:

H1: Brand trust toward agricultural products’ regional public brand is positively correlated with attitude.
H2: Brand trust toward agricultural products’ regional public brand is positively correlated with purchase intention.
H3: Brand trust toward agricultural products’ regional public brand is positively correlated with purchase behavior.
H4: Attitudes toward agricultural products’ regional public brand is positively correlated with purchase intention.

### 2.2 Subjective norms

Subjective norms, the second component of the TPB, encapsulate the perceived social pressure that influences the performance or avoidance of behavior [50]. This pressure may emanate from friends, family, colleagues, media, or propaganda in popular culture [59]. Specifically, word-of-mouth marketing, particularly by friends and family, represents a potent element in the informal communication process between brands and consumers [60]. These unsolicited endorsements can greatly enhance the appeal of branded products. Hence, under the influence of subjective norms, consumers are likely to display heightened positive attitudes toward agricultural products’ regional public brand [61].

Moreover, the surge in social media and live e-commerce platforms has reshaped consumer purchasing behavior toward agricultural products. Live-streaming promotions enable direct seller–consumer interactions, offering in-depth product information and allowing visual inspection, enhancing purchase intention [4]. Furthermore, real-time interactivity on live platforms [62] and social media usage positively influence sustainable purchasing attitudes [63]. Accordingly, we posit the following:

H5: Subjective norms toward agricultural products’ regional public brand are positively correlated with attitude.
H6: Subjective norms toward agricultural products’ regional public brand are positively correlated with purchase intention.

### 2.3 Perceived behavioral control

Perceived behavioral control is the perceived ease or difficulty of performing a behavior, a reaction to experiences and expected future obstacles, and a judgment of whether one’s behavioral ability under the conditions of specific factors allows the performance of a particular behavior [64]. Their overall perceived risk influences consumers’ purchase intention. Perceived risk decreases as the perceived cost of using the various available information in search and risk reduction activities increases. Meanwhile, purchase intention decreases when acceptable risk exceeds perceived risk [65].

As the convenience of e-commerce has increased, the propensity to purchase agricultural products online has risen [3]. Geographic remoteness, as exemplified by locations such as the city of Hulunbuir, thereby ceases to be a hindrance. In the real economy, producers and sellers of agricultural products have recognized the promotional role of e-commerce and live-streaming economy. Many regional governments have also directly participated, establishing e-commerce bases and logistics networks. An integrated retail environment is expanding, enabling customers to navigate among various shopping and delivery channels by providing an omni-channel consumer experience [66]. This fosters greater loyalty toward fresh retailers among consumers [7]. The deployment of agricultural products’ regional public brand, similar to organic certification and geographical indication, can expedite the consumer decision-making process [67]. Consequently, when agricultural products are presented with a regional public brand and provide accessible purchasing channels, consumers’ willingness to purchase is likely to increase. Accordingly, we posit the following:

H7: Perceived behavioral control toward agricultural products’ regional public brand is positively correlated with purchase intention.

### 2.4 Purchase behavior

Purchase intention, a reflection of consumers’ willingness to consume a specific product, is a crucial predictor of purchase behavior [68, 69]. Indeed, purchase behavior is the ultimate economic decision of consumers [57] and the final goal of brand building. While consumers’ intention and behavior vary based on their personalities [5] and individual or family factors [70], the TPB suggests that intention always influences behavior. Such general theories also apply to our research, indicating that a consumer’s purchasing intention towards agricultural products’ regional public brands can facilitate their puchase behavior. Accordingly, we posit the following:

H8: Purchase intention toward agricultural products’ regional public brands is positively correlated with purchase behavior.

**Fig 1:**
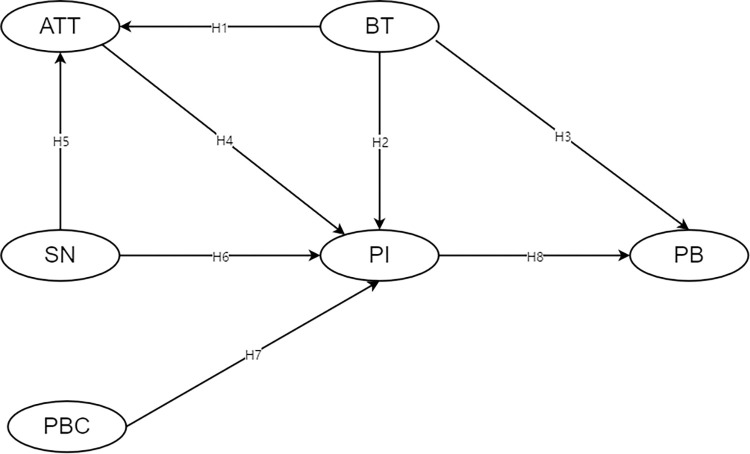
Conceptual model diagram.

The conceptual model is shown in Fig 1.

## 3. Methods

### 3.1 Participants and procedure

This study focuses on Hulunbuir’s agricultural products’ regional public brand. Hulunbuir, a city in the northeastern Inner Mongolia Autonomous Region, China (48°48′-50°12′N, 118°22′-121°02′E), is one of the country’s largest county-level cities, accounting for approximately one-fortieth of China’s total area. It encompasses a diverse range of agricultural products due to its 27 million mu of farmland and 140 million mu of grassland. Through concerted efforts by the government and agricultural producers, today, Hulunbuir hosts eight agricultural products’ regional public brands, including mutton, milk, beef, soybean, canola oil, black fungus, potato, and blueberry brands.

This study collected data through an online questionnaire. The questionnaire respondents were adults (18 or above), capable of making independent purchasing decisions. The questionnaires were subject to pretesting and formal survey phases. During the pretesting stage, we distributed the survey to the participants in a special agriculture product shopping chat group of which one researcher is a member. Before administering the survey, we clearly communicated its scientific research purpose to the participants, assuring them that no personal data would be collected. As the group contained many members, we explicitly requested that only consumers familiar with Hulunbuir respond to the survey. Given that this was the pretesting phase, we ceased data collection immediately after receiving 50 valid responses. The respondents, varying in age, resided in Hulunbuir City, Beijing, and Shandong, China. The feedback from this pretest aligned with our expectations and informed the refinement of our questionnaire for increased clarity and precision.

During the formal phase of the questionnaire, we first revised the text to more explicitly target our theme of Hulunbuir’s agricultural products’ regional public brand, modifying any vocabulary prone to confusion due to English translation. Secondly, we broadened the distribution channels beyond those used in the pretesting phase to various agricultural product purchase chat groups and Hulunbuir tourism discussion forums. The survey was disseminated at different times to diversify the respondents and prevent homogeneity. To ensure that the purpose of the survey was met, we specified in the instructions that only respondents familiar with Hulunbuir should participate. The formal survey was distributed from July to August 2022, during the tourist season, making the Hulunbuir-themed questionnaire appealing to respondents. We successfully collected 569 responses from participants across 27 provinces in China. Following the screening process, we excluded 25 invalid questionnaires due to non-compliance with age specifications and duplications, resulting in a final sample size of 544 and a validity rate of 95.60%.

The questionnaire contained demographic items concerning statistics such as gender, age, monthly income, marital status, and number of family members. The questionnaire also investigated whether the respondents had visited Hulunbuir. A question on purchasing purpose (daily consumption or gift to friends and relatives) was designed. The questionnaire began with a brief introduction of Hulunbuir’s agricultural products’ regional public brand and the logo to ensure that the respondents could understand the survey topic accurately.

As shown in Table 1, a high proportion of respondents were female (70.77%), married (63.60%), 31–40 years old (49.45%), and had more than three family members (70.22%). This distribution aligns with the perspective that females primarily handle household purchases, and that middle-aged consumers who are married and have larger families may show greater interest in the topic of the questionnaire. Since the questionnaire was distributed across several provinces in China, respondents were roughly evenly divided when categorized based on a monthly income of 6000 Chinese yuan. Most of the respondents had visited Hulunbuir (80.51%), which indicates that the respondents were clear about the geographical location of Hulunbuir and could identify Hulunbuir’s agricultural products’ regional public brand. Moreover, a substantial number of respondents had purchased or intended to purchase Hulunbuir regional public brands’ agricultural products for daily consumption (75.92%), which is consistent with the nature of agricultural products.

**Table 1:** The distribution characteristics of the sample.

Statistical characteristic	Type	Frequency	Percentage
Gender	Male	159	29.23%
	Female	385	70.77%
Monthly income	<6000 RMB	262	48.16%
	≥6000 RMB	282	51.84%
Marital status	Married	346	63.60%
	Unmarried	198	36.40%
Age (year)	18–30	78	14.34%
	31–40	269	49.45%
	41–50	99	18.20%
	≥51	98	18.01%
Number of family members	<3	162	29.78%
	≥3	382	70.22%
Whether to visit Hulunbuir	YES	438	80.51%
	NO	106	19.49%
Purpose of purchase	Daily consumption	413	75.92%
	Gift for friends and family	131	24.08%

### 3.2 Measures

To ensure the accuracy of the scale translation, we invited two members from our university’s English department to translate and subsequent back-translation of the scales. All items were rated on a five-point Likert scale ranging from 1 to 5, where 1 means strongly disagree and 5 means strongly agree.

The items on brand trust referred to Zhao et al. (2019) [71] and Konuk et al. (2015) [48], totaling four items. For instance, one item was, “I feel that the agricultural products of Hulunbuir’s regional public brands are trustworthy.”

The items on attitude, subjective norms, and perceived behavioral control mainly followed well-established scales [50, 54]. The items on purchase intention and behavior followed Sun et al. (2022) [72] and Ajzen (2020) [50]. All items were modified to integrate them with the context of this study. Each construct on attitude, subjective norm, perceived behavioral control, purchase intention, and purchase behavior was measured by three items. The scale ultimately consisted of 19 items across 6 constructs. A total of 544 respondents were surveyed, significantly surpassing the guideline minimum of 190. This guideline, as recommended by Kline (2011) [73], suggests a sample size at least ten times greater than the number of measurement items.

## 4. Results

### 4.1 Measurement model

This study used SPSS and Amos software for data analysis. According to Table 2, the Cronbach’s α for each question item in the scale was 0.875, 0.798, 0.810, 0.852, 0.809, and 0.863, signifying robust reliability, as all these values meet or closely approach the recommended value of 0.8 [74]. To test the measurement model, we conducted confirmatory factor analysis (CFA). Each item’s standard factor loading coefficients ranged from 0.726 to 0.902, implying a good measurement relationship. All six constructs corresponded to AVE values were higher than 0.5, and all CR values were higher than 0.7, indicating good convergent data validity.

**Table 2:** Reliability and validity.

Variable	Item	Std. factor loading	Cronbach’s α	CR	AVE
Brand Trust (BT)	BT1	0.798	0.875	0.876	0.639
	BT2	0.853			
	BT3	0.776			
	BT4	0.768			
Attitude (ATT)	ATT1	0.742	0.798	0.799	0.571
	ATT2	0.755			
	ATT3	0.807			
Subjective Norm (SN)	SN1	0.785	0.810	0.812	0.591
	SN2	0.838			
	SN3	0.813			
Perceived Behavioral Control (PBC)	PBC1	0.743	0.852	0.853	0.66
	PBC2	0.796			
	PBC3	0.726			
Purchase Intention (PI)	PI1	0.801	0.809	0.809	0.586
	PI2	0.738			
	PI3	0.757			
Purchase Behavior (PB)	PB1	0.902	0.863	0.867	0.687
	PB2	0.786			
	PB3	0.792			

As shown in Table 3, the model had a good fit (χ^2^/df = 1.869, CFI = 0.975, GFI = 0.975, RMSEA = 0.04, TLI = 0.969, SRMR = 0.038) [75]. According to Table 4, the AVE square root values of all six constructs were higher than the maximum value of the absolute value of the interfactor correlation coefficient, indicating good discrimination validity [76].

**Table 3:** Overall fit indices of the measurement model.

	χ^2^/df	CFI	TLI	RMSEA	SRMR
Scores	1.869	0.975	0.969	0.04	0.038
Criteria	<3	> 0.9	> 0.9	< 0.05	<0.05

**Table 4:** Discrimination validity.

	BT	ATT	SN	PBC	PI	PH
BT	**0.803**					
ATT	0.391	**0.756**				
SN	0.079	0.311	**0.767**			
PBC	0.081	0.088	0.126	**0.814**		
PI	0.321	0.385	0.21	0.363	**0.766**	
PH	0.469	0.246	0.101	0.114	0.392	**0.831**

Note: Each bolded diagonal value represents the square root of the AVE

### 4.2 Hypotheses tests

**Fig 2:**
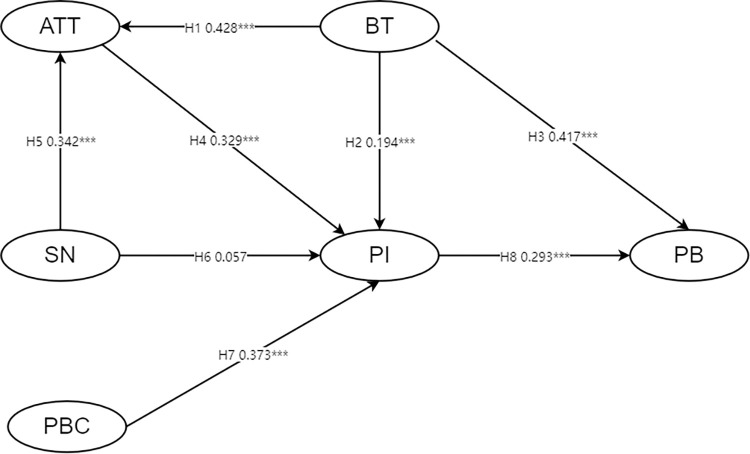
Model framework and SEM analysis results.

SEM allows the elucidation of linear relationships among latent variables, thereby simulating the intrinsic logical interplay among multiple factors [77]. Our eight formulated hypotheses were thus evaluated. The model framework and corresponding path coefficients are presented in Fig 2.

Table 5 summarizes the hypothesis testing. Significant standardized path coefficients supported Hypotheses 1–5 and 7–8. Brand trust toward agricultural products’ regional public brand significantly influences attitude (0.428, p<0.001), purchase intention (0.194, p<0.001), and purchase behavior (0.417, p<0.001), thereby supporting Hypotheses 1 to 3. Attitude toward agricultural products’ regional public brand significantly affects purchase intention (0.329, p<0.001), supporting Hypothesis 4. Subjective norms significantly impact attitude (0.342, p<0.001), supporting Hypothesis 5. Perceived behavioral control significantly affects purchase intention (0.373, p<0.001), supporting Hypothesis 7. Finally, purchase intention significantly influences purchase behavior (0.293, p<0.001), verifying Hypothesis 8. Hypothesis 6 is not supported, as we found that subjective norms do not significantly influence purchase intention (p = 0.26).

**Table 5:** Results of hypothesis testing through SEM.

Path		Estimate	SE	Z	P	Std. Estimate	Result
H1	BT→ATT	0.358	0.042	8.439	***	0.428	Supported
H2	BT→PI	0.197	0.053	3.724	***	0.194	Supported
H3	BT→PB	0.485	0.056	8.609	***	0.417	Supported
H4	ATT→PI	0.399	0.075	5.337	***	0.329	Supported
H5	SN→ATT	0.348	0.052	6.629	***	0.342	Supported
H6	SN→PI	0.071	0.063	1.126	0.26	0.057	Rejected
H7	PBC→PI	0.441	0.056	7.848	***	0.373	Supported
H8	BI→PB	0.336	0.055	6.064	***	0.293	Supported

Note: *** p < 0.001.

### 4.3 Mediation effect

To examine the mediation effect, this study adopted the bootstrap sampling method due to its high acceptance rate in the field. As shown in Table 6, both the “BT→ATT→PI→PB” and “BT→PI→PB” paths were found to be significant (both Bias Corrected and Percentile Method 95% CI did not include zero), suggesting that brand trust toward agricultural products’ regional public brand influences purchase behavior both directly and through these indirect pathways. Purchase intention toward agricultural products’ regional public brand was also identified as a stronger mediator in the relationship between brand trust and purchase behavior.

**Table 6:** Bootstrap mediation effect.

Path	Effect	Bias Corrected (95%)			Percentile method (95%)		
		LLCI	ULCI	P	LLCI	ULCI	P
BT→ATT→PI→PB	0.041	0.023	0.071	***	0.021	0.067	***
BT→PI→PB	0.057	0.027	0.101	***	0.024	0.095	***
SN→ATT→PI→PB	0.033	0.018	0.057	***	0.016	0.054	***
SN→PI→PB	0.017	-0.01	0.052	0.204	-0.013	0.05	0.263
PBC→PI→PB	0.109	0.068	0.156	***	0.066	0.155	***

Note

*** p < 0.001

** p < 0.01

* p < 0.05

In addition, the path “SN→ATT→PI→PB” demonstrated significance (both 95% CIs did not include zero), suggesting that subjective norms toward agricultural products’ regional public brand can indirectly impact purchase intention and behavior via attitude. In contrast, the path “SN→PI→PB” was insignificant (Bias Corrected CI: -0.01, 0.052, 95% CI included zero), confirming our previous rejection of H6.

The path “PBC→PI→PB” demonstrated significance (both 95% CIs did not include zero), indicating that the effect of perceived behavioral control toward agricultural products’ regional public brand on purchase behavior is mediated by purchase intention. Accordingly, by combining these mediating paths, our findings suggest that a combination of factors influences consumers’ ultimate purchase behavior toward agricultural products’ regional public brand.

### 4.4 Multigroup analysis

Personal characteristics lead to consumers’ disparate perceptions of brands, influencing their purchase intention and behavior [70]. Hence, we performed a multigroup analysis to better understand these influences on agricultural products’ regional public brands.

This analysis accounted for gender, age, income, marital status, previous visits to Hulunbuir, and purchasing purposes.

We assessed measurement invariance and multigroup SEM structural invariance following the method proposed by Jaspers and Pearson (2022) [78]. Our analysis was based on seven statistical characteristics collected from our survey: gender, monthly income, marital status, age, number of family members, previous visits to Hulunbuir, and purchasing purposes. These characteristics served as our subgroup criteria for the invariance analyses. In the test of measurement invariance, we conducted both the CFI difference test and the Chi-square difference test to assess the overall model fit between the constrained and unconstrained models for each subgroup [79]. According to Gaskin and Lim (2018) [80] excluding the subgroup “number of family members”, the p-value of the Chi-square difference test was significant (p < 0.05), indicating differences in the model across subgroups. Therefore, we proceeded with the invariance testing of the structural model for the remaining six subgroups, as detailed in Tables 7 and 8.

**Table 7:** Standardized coefficients of the structural model across subgroups: Gender, age, and income.

Path		Gender		Age		Monthly Income	
		Male	Female	<40	≥40	<6000 RMB	≥6000RMB
H1	BT→ATT	0.350***	0.456***	0.425***	0.434***	0.480***	0.386***
H2	BT→PI	0.15	0.215***	0.240***	0.104	0.200*	0.192**
H3	BT→PB	0.245**	0.469***	0.300***	0.582***	0.343***	0.477***
H4	ATT→PI	0.449***	0.275***	0.287***	0.374***	0.350***	0.278***
H5	SN→ATT	0.481***	0.288***	0.378***	0.289***	0.314***	0.352***
H6	SN→PI	-0.04	0.082	0.109	-0.023	0.011	0.089
H7	PBC→PI	0.421***	0.365***	0.374***	0.380***	0.363***	0.389***
H8	PI→PB	0.027	0.379***	0.304***	0.306***	0.145	0.417***

Note

*** p < 0.001

** p < 0.01

* p < 0.05.

**Table 8:** Standardized coefficients of the structural model across subgroups: Marital status, visit to Hulunbuir, and purchase purpose.

Path		Marital status		Vsit to Hulunbuir		Purpose of purchase	
		Married	Unmarried	Yes	No	Daily consumption	Gift
H1	BT→ATT	0.453***	0.352***	0.430***	0.397***	0.446***	0.394***
H2	BT→PI	0.203**	0.174*	0.171**	0.316**	0.163**	0.249*
H3	BT→PB	0.528***	0.212*	0.438***	0.277**	0.428***	0.363**
H4	ATT→PI	0.300***	0.320**	0.331***	0.233†	0.339***	0.349*
H5	SN→ATT	0.330***	0.366***	0.353***	0.304**	0.319***	0.424***
H6	SN→PI	0.016	0.137	0.023	0.250*	0.066	0.008
H7	PBC→PI	0.406***	0.319***	0.388***	0.324**	0.374***	0.355***
H8	PI→PB	0.276***	0.291**	0.200***	0.636***	0.320***	0.185

Note

*** p < 0.001

** p < 0.01

* p < 0.05.

Tables 7 and 8 demonstrate subgroup noninvariance. Within the gender subgroup, the paths “BT → PI” and “PI → PB” were significant exclusively for females, with the path “BT → PB” demonstrating a stronger effect among females. This conclusion is somewhat influenced by the larger proportion of females (70.77%) in the sample. Conversely, the “SN → ATT” path was more robust for males. Age-related differences were observed in the impact of brand trust toward agricultural products’ regional public brand on purchase intention and behavior, with the “BT → PI” path significant only for respondents under 40 and the “BT → PB” path stronger for those aged 40 and above.The paths “BT → PB” and “PI → PB” were stronger for respondents with a monthly income exceeding 6000 RMB.

For the marital status subgroup, the “BT → PB” path was stronger for married people, which is also somewhat influenced by the distribution of the sample. Regarding the visiting experience, the “BT → PB” path was stronger for those who had visited Hulunbuir, while the “PI → PB” path was more pronounced for those who had not. Based on the above rejection of Hypothesis 6, we excluded the “SN → PI” path from this analysis. In the purpose subgroup, the “PI → PB” path was significant only for daily consumption. Among the six subgroups with noninvariance, the relationship between brand trust and purchase behavior toward agricultural products’ regional public brand was influenced in five of them. Finally, the remaining hypothetical paths were invariant.

## 5. Discussion and implications

### 5.1 Discussion

In our study, we evaluated eight hypotheses using SEM, based on the analysis of 544 valid questionnaire responses. We concentrated on the, thereby expanding the scope of the research. We employed mediation analysis to explore the indirect effects within our model, and conducted multigroup analysis to identify the influence of different group factors. Beyond these empirical findings, our study also contributes to the existing literature in several ways.

First, by constructing a model that integrates brand trust and the TPB, we find brand trust in agricultural products’ regional public brand positively affects attitude, purchase intention, and purchase behavior. In terms of purchase behavior toward agricultural products’ regional public brand, brand trust is the most influential factor among the direct influences. Each regional public brand, with its unique characteristics, integrates certification and location factor of geographical indication, and sends a positive and guaranteed signal to consumers. This heightens consumer affinity and identification with agricultural products and facilitates the transformation of purchase intention into actual purchase behavior [81]. While brand trust directly influences purchasing behavior, it can also affect it indirectly via its impact on attitude and purchase intention. This finding is consistent with the original purpose of China’s regional public branding, emphasizing the importance of brand operation [82], which is also the highlight of this study.

Second, according to TPB, a key component of this study’s theoretical framework, both attitude and perceived behavioral control toward agricultural products’ regional public brand positively influence purchase intention. When consumers form a favorable attitude toward a product’s regional public brand, they actively promote it and convert it into purchase behavior. This conclusion aligns with research findings on purchase intention regarding green and organic food products [22, 72].

Perceived behavioral control toward agricultural products’ regional public brand has a more significant effect on purchase intention than attitude. This discrepancy primarily arises from the focus of our study on Hulunbuir, a relatively remote region in China. Consumers hence need to consider the feasibility of purchasing branded agricultural products from this area, which can lead to conclusions that vary slightly from those in broad-based agricultural research [83, 84]. Facilitated by e-commerce platforms, live-streaming product recommendations, and advancements in logistics, consumers now have more purchasing channels and shorter time frames to acquire desired products than in the past. Transparency in agricultural product pricing, a result of market competition, further simplifies the buying process for consumers [11]. Consequently, consumers can have enhanced experiences when purchasing branded agricultural products directly from their places of origin, leading to their increased willingness to buy and actual purchase behavior [85].

As for subjective norms toward agricultural products’ regional public brand do not directly influence consumers’ purchase intention but can shape their attitude [86]. This may be because consumers have well-formed consumption views, making them less affected by the purchasing behavior of friends or relatives and media advertising. However, these external influences can strengthen consumers’ positive attitude toward a product and indirectly influence their purchase intention and behavior. Therefore, subjective norms cannot be ignored in studies of consumers’ purchase intention and behavior.

Thirdly, multigroup analysis further highlights the influences exerted by different factors. We have found that consumers’ characteristics also impact their purchase intention and purchase behavior regarding agricultural products’ regional public brands. While female consumers maintain their enthusiasm after trying branded products [87], an interesting finding in this study is that male consumers tend to have a more favorable attitude toward regional public brands due to external promotion and their friends’ recommendations. Consumers over the age of 40 are also likely to directly purchase branded products, a behavior indicative of impulse buying influenced by a brand’s presence [88]. For those under 40, Generation Y’s shopping is based on their choice of products [89]. Furthermore, the influence of consumers’ income on their purchase behavior is immeasurable [90]. Consumers with a monthly income of more than RMB 6,000 are the most likely to transform their intention into actual purchase behavior towards agricultural products with regional public brands. Married consumers tend to show stronger support for a brand and are likely to make purchases based on trust. Similarly, consumers who have visited Hulunbuir exhibit a higher likelihood of purchasing agricultural products with a regional public label, reflecting their trust in their brand. Consumers who regularly purchase agricultural products for everyday consumption are more likely to transform their purchase intention into actual purchase behavior, consistent with the typical use of agricultural products.

### 5.2 Practical implications

First, the owners and operators of an agricultural product’s regional public brand must prioritize maintaining and enhancing consumers’ trust in their brand. Trust in a brand requires a long-term strategic marketing approach, including brand building, fostering brand equity, and implementing ongoing supervision. Currently, most agricultural product’s regional public brand are established and hosted by regional governments. These governments can provide government-initiated innovations and financial support to enhance their agricultural products’ branding effect [91]. Beyond traditional celebrity endorsements, government officials have personally participated in brand promotion. Brand equity is collectively built by numerous licensed, qualified agricultural operators. As a result, brand image must be jointly protected by stakeholders. In particular, many agricultural operators should maintain self-discipline, be patient enough to realize long-term benefits, eliminate free-riding, and work together to improve their quality assurance system and realize the traceability of food to its source. Ongoing supervision of agricultural product’s regional public brand is also crucial. If brands are abused, a scenario known as “greenwashing” may occur, leading to consumers distrusting all the products associated with such brands [39].

Second, enhancing brand trust and integrating it with omnichannel marketing is crucial, especially for remote locations such as Hulunbuir. As our research has shown, perceived behavioral control significantly influences purchase intention. Thus, agricultural product operators should enhance consumers’ purchasing assurance through omnichannel marketing. Additionally, local governments where these agricultural products are based should also promote the development of transportation and other infrastructures. For instance, our research focuses on Hulunbuir, in northeast China, where the transportation routes for agricultural products are lengthy. Products like beef and mutton require frozen transportation, while others milk, blueberries, and potatoes require fresh transportation, each presenting unique difficulties.

Third, brand owners and operators should formulate highly targeted marketing strategies and employ targeted advertising to influence individual consumers’ characteristics [92]. Currently, consumers exhibit increased rationality [93]. Consumers with diverse genders, ages, income levels, and marital statuses have unique purchasing preferences. A simple recommendation does not directly convert into a purchasing desire. Word-of-mouth marketing will thus only influence consumers’ attitude when it reaches a certain level of intensity [94]. For instance, with Generation Y gradually becoming the primary consumer group, the novelty-oriented characteristics of this younger generation should be considered.

## 6. Conclusion

This study adopts the TPB framework and incorporates brand trust to investigate the factors influencing consumers’ purchase intention and behavior concerning agricultural products’ regional public brands in Hulunbuir, China. Using a questionnaire survey method and SEM, we examined the effects of various factors. The study also employed mediating effects and multigroup analysis to identify key influencing elements.

The results underscore the significant role of brand trust in shaping attitudes, purchase intentions, and behavior. Furthermore, both attitude and perceived behavioral control were found to exert influence on purchase intentions, with the latter having a more substantial effect. Notably, subjective norms influenced attitudes but did not directly impact purchase intentions. Additionally, consumer characteristics, such as gender, age, monthly income, marital status, previous visits to Hulunbuir, and purchase purpose, were found to significantly influence purchase intention and behavior. These findings highlight the complex influences on consumer behavior towards regional public brands of agricultural products.

Agricultural products’ regional public brand is a marketing concept with extensive coverage. This study has primarily focused on how brand trust, attitude, subjective norms, and perceived behavioral control impact consumer purchase intention and behavior by employing the classic TPB framework. However, many potential factors have not been addressed. Future research could therefore benefit from incorporating additional models to explore further the role of brand trust and other determinants of consumer purchase decisions.

In this study, Hulunbuir’s agricultural products’ regional public brand was the focus of questionnaire research. This sample’s representativeness may be limited. Future studies could gain more comprehensive insights by comparing multiple brands or examining the same brand at different times to identify the strengths and weaknesses of various brand-building processes. Furthermore, the sample for this study was randomly distributed, without stringent control for many demographic variables, such as the male-to-female and marital status ratio. Future research could exercise more precise control over the sample according to the demographic characteristics of the research subjects.

## Supporting information

S1 Data (XLSX)

S1 File (DOCX)
