# Supplementary material for: The impact of brand trust on consumers’ behavior toward agricultural products’ regional public brand
Source: PLoS One. 2023 Nov 30;18(11):e0295133. doi: 10.1371/journal.pone.0295133 (PMC10688752; doi:10.1371/journal.pone.0295133)
Supplement: S1 File — (DOCX) [file pone.0295133.s002.docx]

**Annexure 1：Questionnaire items**

**Brand trust toward agricultural products’ regional public brand：**

I feel that the agricultural products of Hulunbuir’s regional public brands are trustworthy.

I feel that the quality of agricultural products of Hulunbuir’s regional public brands is reliable.

I feel that the quality of agricultural products of Hulunbuir’s regional public brands is stable.

The Hulunbuir’s regional public brands keep promise of providing high-quality food from the place of origin.

**Attitudes toward agricultural products’ regional public brand：**

It is a good idea to buy agricultural products of Hulunbuir’s regional public brands.

It is interesting to buy agricultural products of Hulunbuir’s regional public brands.

I have a positive attitude towards buying agricultural products of Hulunbuir’s regional public brands.

**Subjective Norm toward agricultural products’ regional public brand：**

My family and friends favor buying agricultural products of Hulunbuir’s regional public brands.

People whom I value often buy agricultural products of Hulunbuir’s regional public brands

Exposure to media reports and live streaming content would influence my decision to buy agricultural products of Hulunbuir’s regional public brands.

**Perceived Behavioral Control toward agricultural products’ regional public brand：**

If it is entirely up to me, I am confident about buying agricultural products of Hulunbuir’s regional public brands.

There are many channels, and easy to agricultural products of Hulunbuir’s regional public brands.

I have the resources and time to identify and buy agricultural products of Hulunbuir’s regional public brands.

**Purchase Intention toward agricultural products’ regional public brand：**

If I can, I would like to buy agricultural products of Hulunbuir’s regional public brands.

I would try to buy agricultural products of Hulunbuir’s regional public brands.

I would like to recommend agricultural products of Hulunbuir’s regional public brands.

**Purchase Behavior toward agricultural products’ regional public brand：**

I buy agricultural products of Hulunbuir’s regional public brands if I have enough time, energy, and money.

I will prioritize buying agricultural products of Hulunbuir’s regional public brands when I have the same choice.

I am willing to buy agricultural products of Hulunbuir’s regional public brands as gifts for festivals or special occasions.
